# Deepfake anomaly detection using kernel density estimation based convolutional autoencoder

**DOI:** 10.3389/frai.2026.1834939

**Published:** 2026-09-30

**Authors:** Manas Ranjan Prusty, Sahil Amritkar, Ananthakrishnan Balasundaram, Subhra Rani Patra

**Affiliations:** 1 Centre for Cyber Physical Systems, Vellore Institute of Technology, Chennai, India; 2 School of Computer Science and Engineering, Vellore Institute of Technology, Chennai, India; 3 Department of Information Systems and Operations Management, University of Texas at Arlington, Arlington, TX, United States

**Keywords:** anomaly score, convolutional autoencoder, deepfake detection, kernel density estimation, visual anomaly detection

## Abstract

The rise of deepfake technology has fuelled the need for robust detection methods to counter the increasing sophistication of manipulated multimedia content. Research has shown that humans struggle to identify deepfakes, highlighting the pressing need for AI-based solutions to safeguard the integrity of the information we consume daily. This paper proposes a novel approach CAE-DAD (Convolutional Autoencoder Deepfake Anomaly Detector) which facilitates deepfake detection through visual anomaly identification using convolutional autoencoders (CAEs). Methods like Kernel Density Estimation are used in the latent space to widen the anomaly score gap between real and fake images. The model is trained only on real face images. Existing models like VGG16 which are known for their feature extraction capabilities as incorporated as part of our encoder. The model is benchmarked against state-of-the-art methods and establish a comprehensive set of evaluation metrics, including accuracy, precision, recall, F1 score. The model achieves comparable performance to existing two-class training methods, and achieves 91% accuracy. The proposed anomaly identification using CAEs serves as a valuable addition to the arsenal of tools aimed at mitigating the risks associated with the proliferation of deepfake content.

## 1 Introduction

Deepfakes are forms of media that have been digitally forged to resemble another person, usually a public figure to spread misinformation or out of malice. Deepfakes can be of various forms like videos, images or audio. There are lots of ways to create deepfakes, it can be as simple as using editing software like photoshop, or using complex deep learning models like GANs and autoencoders. Deepfakes pose a multifaceted threat, enabling the spread of misinformation and political manipulation. They compromise privacy, fostering identity theft and unauthorized access. The technology erodes trust in digital media, making it challenging to distinguish authentic content. Legal frameworks struggle to keep pace, contributing to ethical dilemmas. Deepfakes also facilitate cyberbullying, with potential societal consequences. Their ability to deepen existing divides and complicate verification processes in journalism further underscores their impact. Detection challenges exacerbate the problem, necessitating a comprehensive approach to address the ethical, legal, and societal implications of deepfake technology.

The deficiency in humans to identify deepfakes, as deepfake creation technology gets better is highlighted in (Bray et al., 2023). This study aims to assess human ability to identify image deepfakes of human faces curated from the StyleGAN2 algorithm as trained on the FFHQ dataset (Karras et al., 2019). Granted the dataset of images used is small and so is the sample pool of people, but the participant accuracy was 62% overall, this accuracy across images ranged between 30 and 85%. Overall the accuracy was just slightly better than a coin toss. Equally concerning was the participants’ high confidence in their answers. This shows us the need for AI models that can do a much better job. To address this problem, there is a lot of research going on for creating deepfake detection models, some of which are discussed are in the related works section. Most of the approaches for deepfake detection use deep CNN models. These models are excellent at doing what they are trained to do, but cannot be generalized. This paper proposes a different way to think about catching deepfake images, focusing on faces. Autoencoders are used for anomaly detection in a multitude of scenarios, be it time-series data, or other visual anomaly detection scenarios like cracks on walls or medical image diagnosis. We suggest applying this property of autoencoders to catch deepfake images, as deepfakes are nothing but anomalies. At first glance, they look like just any other images, but look a little deeper and the flaws start to emerge.

The motivation behind this work stems from the growing inability of humans to accurately distinguish deepfake images, especially as generative models become more sophisticated. This poses a serious threat across multiple domains, from misinformation to identity theft. The key contribution of this paper is the proposal and development of CAE-DAD, a convolutional autoencoder-based model tailored for deepfake image detection using anomaly detection principles. Unlike existing CNN-based classifiers that rely on learning specific patterns, our approach reframes the problem by leveraging the generalizability of autoencoders to detect subtle irregularities characteristic of deepfakes. The novelty of this work lies in its reinterpretation of deepfakes as visual anomalies and applying autoencoders, commonly used in fields like fault detection and medical imaging for the task of deepfake identification. This perspective opens a new direction in building more robust and adaptable detection systems.

## 2 Related work

An overview of the recent publications and research work done on deepfake detection is shown in (Rana et al., 2022). It shows the most promising and popular technologies/models, datasets, metrics etc. being used by researchers. It compiles the recent trends for us to see, and draw inspiration and guidance for the direction of future work. The paper highlights how deep learning based methods are the best for deepfake detection as compared to statistical, blockchain or machine learning approaches and that majority of the papers use a traditional CNN based approach. A deepfake predictor (DFP) is proposed in (Raza et al., 2022), which is based on the VGG16 architecture. It achieves 94% accuracy on the Real and Fake Face Detection Dataset (Real and Fake Face Detection, 2019). This approach outperforms existing models from 2020–21 in terms of accuracy. They also compare their model with existing systems and achieve better results. The paper does not provide information about the limitations of the proposed deepfake detection approach or any potential challenges faced during the research process.

A Texture-based Local Binary Patterns along with a CNN based modified High-Resolution Network (HRNET) and Capsule Neural Networks implementing a concurrent routing technique is shown in (Khalil et al., 2021). It uses the Celeb-DF (Li et al., 2020) and DFDC-P (Dolhansky et al., 2020) datasets. It achieved and average 12% improvement in frame level scores compared to other models. It also has some generalization capabilities. But capsule networks have a higher implementation complexity when compared to CNNs. A fast and novel hybrid MLP-CNN model is proposed for detecting deepfakes in (Kolagati et al., 2022). It uses the DFDC dataset. The proposed model in the paper achieves an accuracy of 84% and an AUC score of 0.87. The hybrid system offers a great baseline for screening deepfakes with limited computational resources at a relatively faster speed. Due to limited computing resources, the entirety of the DFDC dataset was not used. The proposed system also does not detect faces properly in dark lighting conditions. Further, the system did not take into account multiple people in frame. The aim is to improve the detection of low quality compressed deepfake images in (Le and Woo, 2021). They introduce an Attention-based model for detecting deepfakes, comprising two key components. The first is a frequency attention distillation, which isolates high-frequency components excluded from the student network. The second component is a multi-view attention distillation, which generates multiple attention vectors by slicing the tensors of both teacher and student networks from different perspectives. This approach facilitates a more efficient transfer of the teacher tensor’s distribution to the student network. The method achieves superior performance on compressed (c40) datasets. This method however requires a large memory buffer.

A Vision Transformer (ViT) and CNN based method is proposed in (Coccomini et al., 2022). They compare the model to EfficientNetV2 on the ForgeryNet dataset (He et al., 2021). It says EfficientNetV2 tends to specialize more, often achieving superior performance on training datasets. In contrast, Vision Transformers demonstrate a higher level of generalization, making them more adept at handling images generated with new methodologies. ViT shows closer accuracies on different and unseen deepfake generation methods, making it suitable for real-world applications. The research does not discuss the computational resources required for training and deploying the deepfake detection models, which could be a potential limitation in real-world applications. ResNext, a CNN algorithm and LSTM is used as an approach to detect deepfakes is proposed in (Vamsi et al., 2022). They use the Celeb-DF v2 dataset and achieve a 91% accuracy. The model beats previous works and gets a respectable score on a well-known dataset. The results have been obtained on cheap hardware (Google Colab free tier). However, better models have been developed since. A frequency based CNN is used in (Kohli and Gupta, 2021). They achieve a 66.5% accuracy on the Celeb-DF v2 dataset. The fCNN architecture is only a shallow three convolutional layered design, performing slightly worse to the XceptionNet (a 36 convolutional layered deep architecture) and GAEL Net (a GAN-based architecture). The limitation of the proposed method is its non-uniform facial manipulation detection accuracy, particularly for neural texture facial manipulation.

A deep sparse autoencoder is suggested in (Balasubramanian et al., 2022) for effective feature selection. They use the FF++ (Rössler et al., 2019) and DFDC datasets. Approach has secured an improved accuracy of 98.7, 98.5% for the Face2Face and FaceSwap in FF++, and 97.63% in the DFDC datasets. The paper concludes that the proposed deep learning model yields improved feature extraction performance, thereby mitigating overfitting issues in deepfake detection. The complexity of the proposed deep learning model may require significant computational resources and time for training and optimization, which can be a limitation in real-time applications. An SVM based method is used in (Agarwal et al., 2021). Frequency domain analysis is done after which a classifier will be used to differentiate the real and fake image. It is observed that the 1D spectrum of the images belonging to the same set (real and fake) have similar patterns. It achieves an accuracy of 99.76% on the CelebA (Liu et al., 2014) dataset. The advantage of the proposed method is that it shows good accuracies with fewer amounts of training data. However the dataset may not be complex enough to challenge the model. OC-FakeDect (Khalid and Woo, 2020) is another method that uses only real images to train their model. They use a variational autoencoder and, train and test their model on the FF++ dataset. They develop two models, one which uses the RMSE error of the input and reconstructed image, and another which passes the reconstructed image through the encoder again, and compares the RMSE error of the two latent spaces. The second approach gives slightly better results, but both models perform well and produce 71.20 to 98.20% accuracy on the different subsets of the FF++ dataset. They also test their model against a simple convolutional autoencoder (OC-AE), and say OC-AE does not perform well because it only learns to reconstruct images, and not any features.

A Resnet18 for feature extraction and SVM or KNN for classification on ELA of the data is proposed in (Rafique et al., 2023). They use the real and fake face detection dataset for their work. They achieved 89.5% accuracy using their approach. They also tried using GoogLeNet and SqueezeNet for feature extraction but Resnet18 gave the best results. The proposed methods in (Qurat-ul-ain et al., 2021) are ELA followed by CNN models like VGG16, VGG19, ResNet50 and Inception v3. They achieved the best results using VGG16 on the real and fake face detection dataset, which was 64%. However, there is a big difference between the training and testing accuracy in their model, 92% for training and 64% for testing. These following papers propose various autoencoders for visual anomaly detection. They explore techniques to increase the gap between anomaly scores of the normal and anomaly images. Huang, et al. (2022) proposes reconstructing both the image and its transformation to compute the anomaly score. Ravi and Karray (2021) suggests focus on the reconstruction error based method of AD using CAEs that learn the common attributes of variation from normal data to reconstruct them perfectly. Zhu et al. (2021) suggests using two parallel decoders for image reconstruction, and combining the reconstruction losses for the anomaly scores. Chen et al. (2021) suggests a hashing memory module between the encoder and decoder to produce higher reconstruction error for classification. Cheng et al. (2021) suggests detecting the anomalies in the feature space rather than use reconstruction loss as an anomaly score.

Diffusion models have been extensively used recently to synthesize images. Latent diffusion models used in (Rombach et al., 2022) uses cross attention layers into the model architecture that converts diffusion models into powerful generators. Cycle-LVDM (Cycle-Consistent Latent Variational Diffusion Model) (Kui et al., 2026) uses an unpaired latent variational diffusion framework for medical image translation. Partitioned latent diffusion model (p-LDM) (Kim et al., 2026) partitions 3D images and translates them in latent space efficiently. p-LDM that reduces GPU memory usage while preserving translation quality. Semantic-guided adaptive latent space truncation sampling for watermarking of diffusion models (Guo et al., 2026) constructs semantic importance maps via CLIP-guided activation scores. It introduces adaptive truncation sampling with dynamic thresholding strategies.

## 3 Proposed methodology

The architecture of the system has been built by starting with a base that has seen success in other similar applications and by tweaking the layers and parameters by trial and error to see what works for our application.

The overall architecture of CAE-DAD is shown in Figure 1. It explains the three phases of the model: training, threshold setting, and classification. The images are cropped with RetinaFace (Poojary et al., 2021). The convolutional autoencoder is optimized on real image data until the reconstruction loss falls below a predefined threshold, indicating satisfactory learning. Then the reconstruction loss (MSE) is taken, and its distribution is observed. Based on it, a threshold value is chosen. The Kernel Density Estimation (KDE) model is fitted to the real training images latent spaces, and the distribution is observed. A threshold value is chosen for KDE too. Now for testing when an image is fed into the autoencoder, it will reconstruct it based on its learning of encoding and reconstructing real images. The latent space of the test image is also fed into the KDE model for sampling. If it is a real image, the MSE and KDE values will be in the expected range, but if it was a deepfake, it would be an anomaly and the MSE value would be above the threshold value, or the KDE value would be below the threshold and would be classified as such. The main purpose of the model is to extend this gap between the MSE values of reconstructed real and reconstructed fake images.

**Figure 1:**
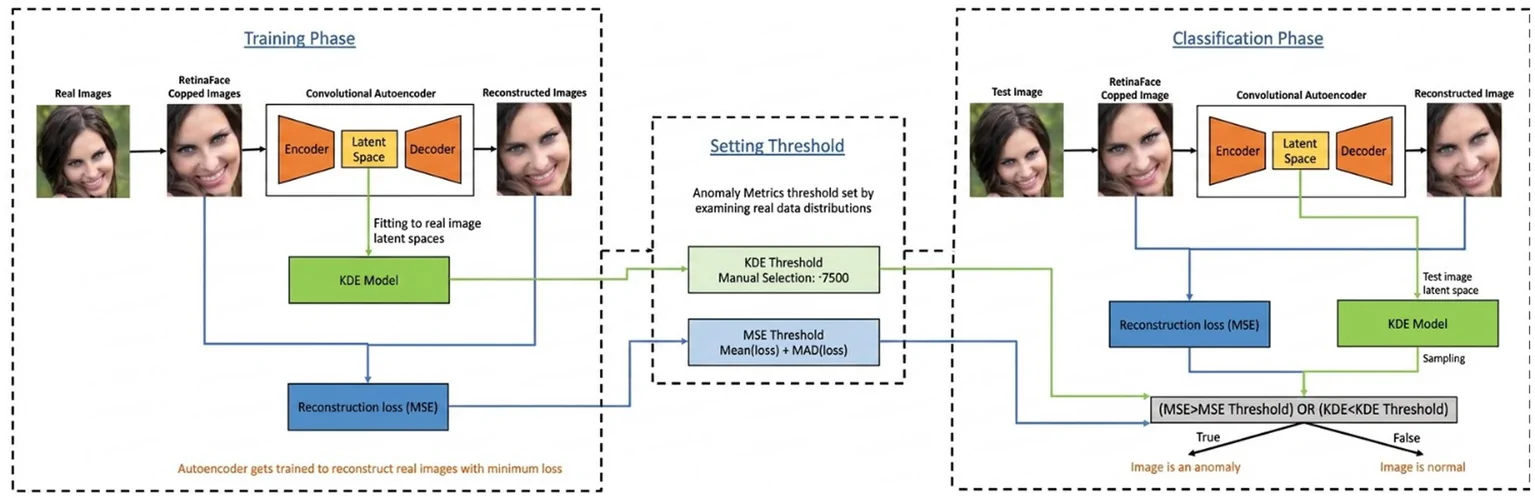
Architecture of CAE-DAD (images adapted from the Real and Fake Face Detection Dataset, https://www.kaggle.com/datasets/ciplab/real-and-fake-face-detection, licensed under CC-BY-NC-SA 4.0).

### 3.1 Data augmentation

As the dataset used is small, data augmentation is needed to ensure a robust model that does not overfit on the limited training data. The benefits of using data augmentation with a CNN model, especially a VGG16 based one are shown in Poojary et al. (2021). They use transfer learning on VGG16 and see an increase of around 8% in accuracy. We perform the following data augmentations on the training data. The brightness is altered by ±10%. The zoom level is also altered by ±10%. Horizontal Flipping is also enabled. All these alterations will expand the dataset enough such that the chances of overfitting are decreased, but still retain the characteristics of real face images so features can be learnt. Figure 2 shows the effects of these transforms on a few sample images.

**Figure 2:**
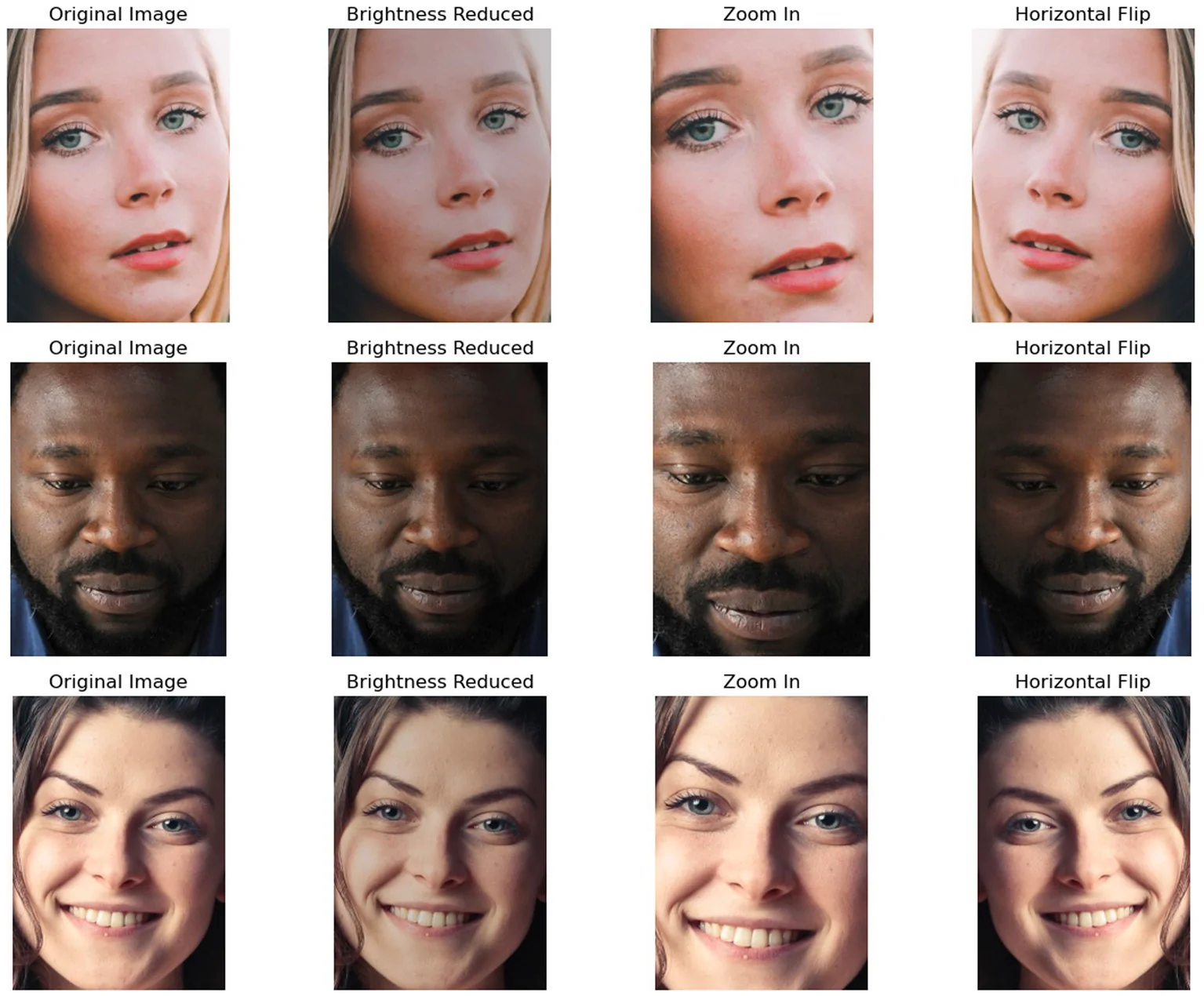
Effects of various transforms used on sample images (images adapted from the Real and Fake Face Detection Dataset, https://www.kaggle.com/datasets/ciplab/real-and-fake-face-detection, licensed under CC-BY-NC-SA 4.0).

### 3.2 Convolutional autoencoder (CAE)

The convolutional autoencoder is made up of three main parts, the encoder, the latent space, and the decoder. During training the model updates its weights to lower the reconstruction MSE loss. While predicting, the model uses these weights to encode to reconstruct the input image. Figure 3 shows the architecture of the convolutional autoencoder. The encoder consists of an input layer, a VGG16 (Simonyan and Zisserman, 2014) functional layer, a batch normalization layer, a flattening layer and finally a dense layer. VGG16 has been chosen as it is known for its feature extraction capabilities, and has been used previously in facial recognition models like VGGFace (Parkhi et al., 2015). VGG16 turns the (128,128,3) image into a tensor of (4,4,512) after performing feature extraction. The flattening layer turns this three-dimensional tensor into a vector of size 8,192, and a dense layer then further compresses it down to a vector of size 4,096. Batch Normalization speeds up the training process by reducing the sensitivity to initial weights. The latent space is the compressed form of the original image, from which the decoder must learn to reconstruct the images. The latent space is a 1D vector of size 4,096. A flattened latent space with dense layers gave better results than a 3D latent space. KDE also requires a flattened vector input. The decoder has a dense layer and a reshape layer to bring the output back to a three-dimensional tensor from the flattened vector. This is followed by 4 sets of up-sampling, conv2D and batch normalization layers, which take the tensor from a shape of (4,4,512) to a shape of (64,64,32). Finally, an up-sampling layer and a Conv2D layer transform the tensor back to the original image size of (128,128,3).

**Figure 3:**
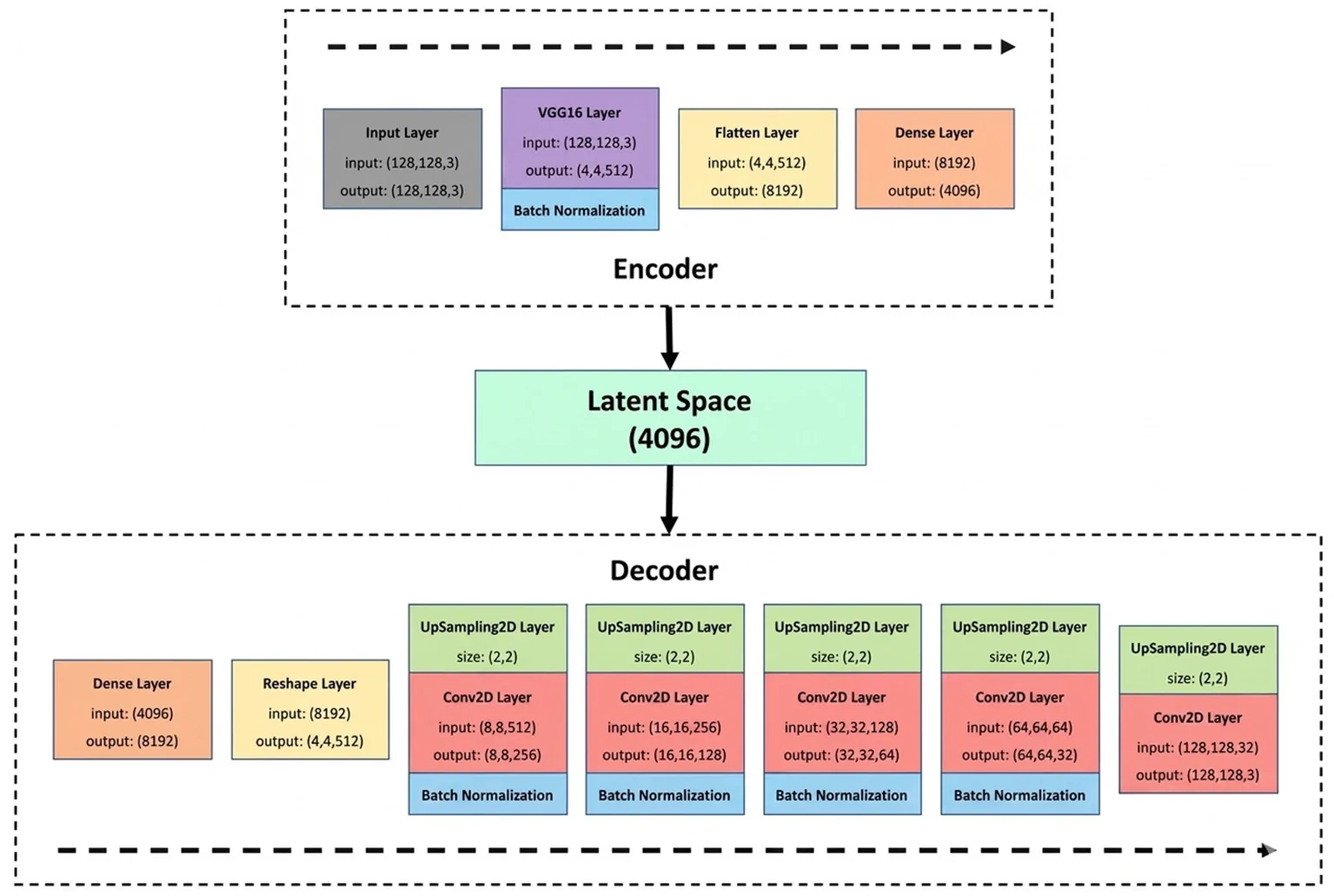
Architecture of the convolutional autoencoder.

### 3.3 Training

The convolutional autoencoder is trained only on real images. Adam (Kingma and Ba, 2014) is used as the optimizer and MSE as the loss for the model. The initial weights of the model are set to random. The model is trained for 150 epochs, when convergence is reached. The model is trained until it achieves a satisfactory reconstruction accuracy on real images.

### 3.4 Face cropping

Face cropping is used as a way to add attention to our model. Having a cluttered background reduces the ability of a model to focus on the important parts, which in this case is the face. The improvements seen by using a way to remove a cluttered background are explained in (Kc et al., 2021). A decoder will not be able to reconstruct every background perfectly, which will lead to a lower MSE and skew the results, hence it is best to completely remove it and isolate the face. This allows the model to focus on the face, no matter the context, and can be used for both, training images and testing images. RetinaFace (Deng et al., 2020) is used to find the coordinates of the face from the image, and then crop them. Figure 4 demonstrates how a photo is cropped using RetinaFace face detection. The left column shows the original image. The middle column uses the green rectangle to show the coordinates of the face returned by RetinaFace. The final column shows the face image after being cropped. The performance benefit of using RetinaFace cropping is shown in section 5.3.

$$\text{cropped}\_\mathrm{img}(i,j)=\mathrm{img}(y_{1}+i,x_{1}+j)$$ (1)

**Figure 4:**
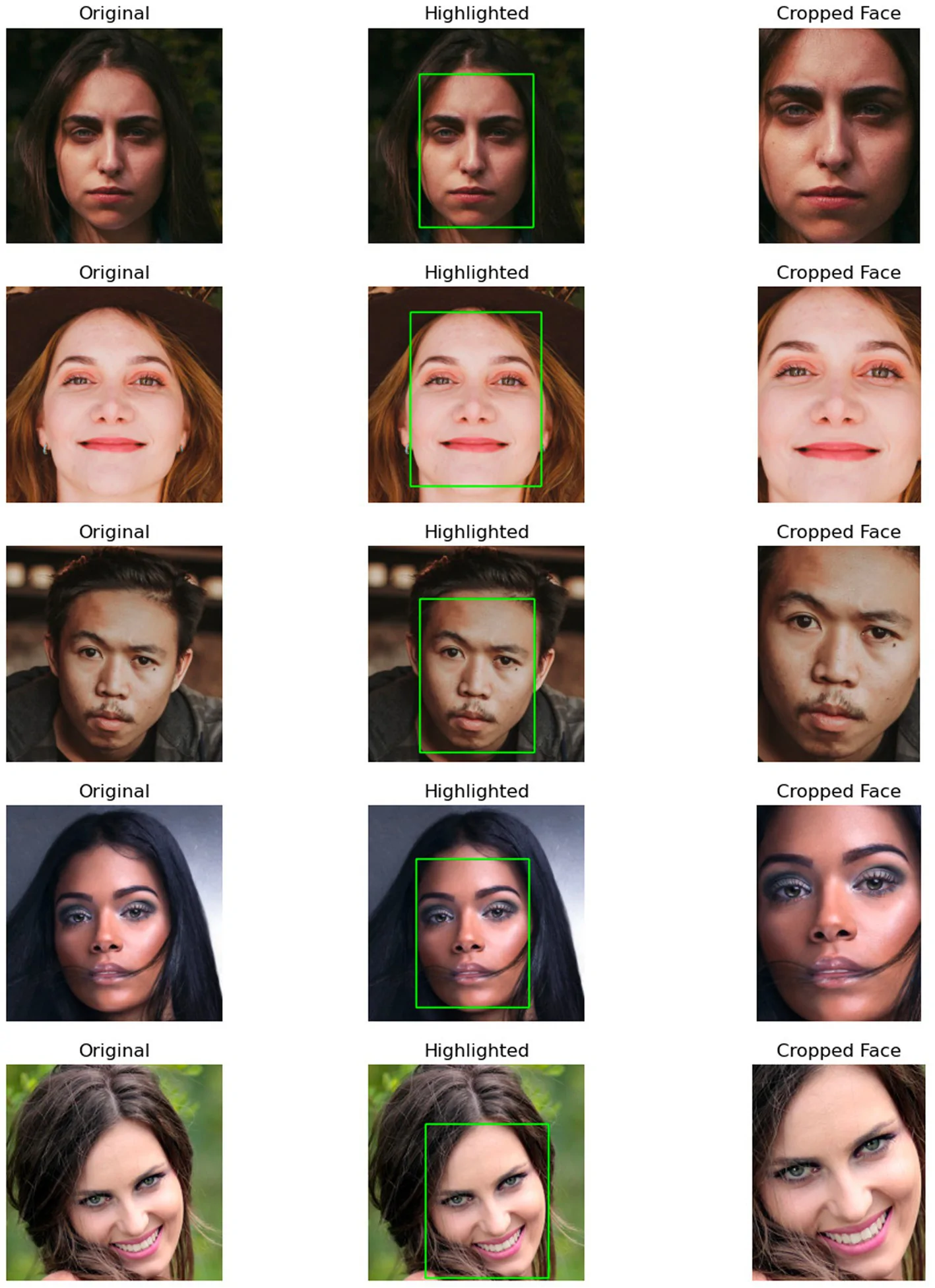
Demonstrates the face cropping process (images adapted from the Real and Fake Face Detection Dataset, https://www.kaggle.com/datasets/ciplab/real-and-fake-face-detection, licensed under CC-BY-NC-SA 4.0).

The equation for cropping the image is shown in Equation 1. The cropped image is obtained by selecting a rectangular region of interest specified by the coordinates *(* $x_{1},y_{1}$ *)* (top left corner) and *(* $x_{2},y_{2}$ *)* (bottom right corner) from the original image. Hence *i = 0,1,…, (* $y_{2}-y_{1}$ *)* and *j = 0,1…,(* $x_{2}-x_{1}$ *).*

### 3.5 Anomaly metrics

We use two anomaly metrics, mean squared error (MSE) between the original and reconstructed image, and kernel density estimation (KDE) of the latent space. We explain both in detail below.

In the context of comparing two images, Mean Squared Error (MSE) can be used to measure the average squared difference between corresponding pixel values of the two images. It is a common metric used in visual anomaly detection through autoencoders, like for example in (Siddalingappa and Kanagaraj, 2021). The general equation for MSE is given by Equation 2. The equation for finding the MSE error between two RGB images is given by Equation 3, where *m* and *n* are the dimensions of the image, and *(i,j)* are the pixel coordinates of an image. *R*, *G*, and *B* represent the red, green, and blue channels respectively, and *I_1_* and *I_2_* are the two images.

$$\mathrm{MSE}(X,Y)=\frac{1}{n}\sum_{i=1}^{n}{\left(X_{i}-Y_{i}\right)}^{2}$$ (2)

$$\mathrm{MSE}(I_{1},I_{2})=\frac{1}{3\mathrm{mn}}\sum_{i=1}^{m}\sum_{j=1}^{n}(\begin{array}{l} {\left(I_{1}^{R}(i,j)-I_{2}^{R}(i,j)\right)}^{2}\\ +{\left(I_{1}^{G}(i,j)-I_{2}^{G}(i,j)\right)}^{2}\\ +{\left(I_{1}^{B}(i,j)-I_{2}^{B}(i,j)\right)}^{2} \end{array})$$ (3)

The MSE reconstruction values are recorded for the training real images, testing real images, and testing fake images is shown in Figure 5. The average value line is calculated for all the samples, and 100 random samples are plotted from all three distributions. The key to the model’s success is maximizing the gap between the real and fake distributions. The lesser the overlap between the distributions, the better the efficacy of the threshold and higher will be the accuracy. As seen in graphs, the distribution and average MSE of the testing real images is close to the training real images, while there is a clear distinction between the real and fake distributions.

**Figure 5:**
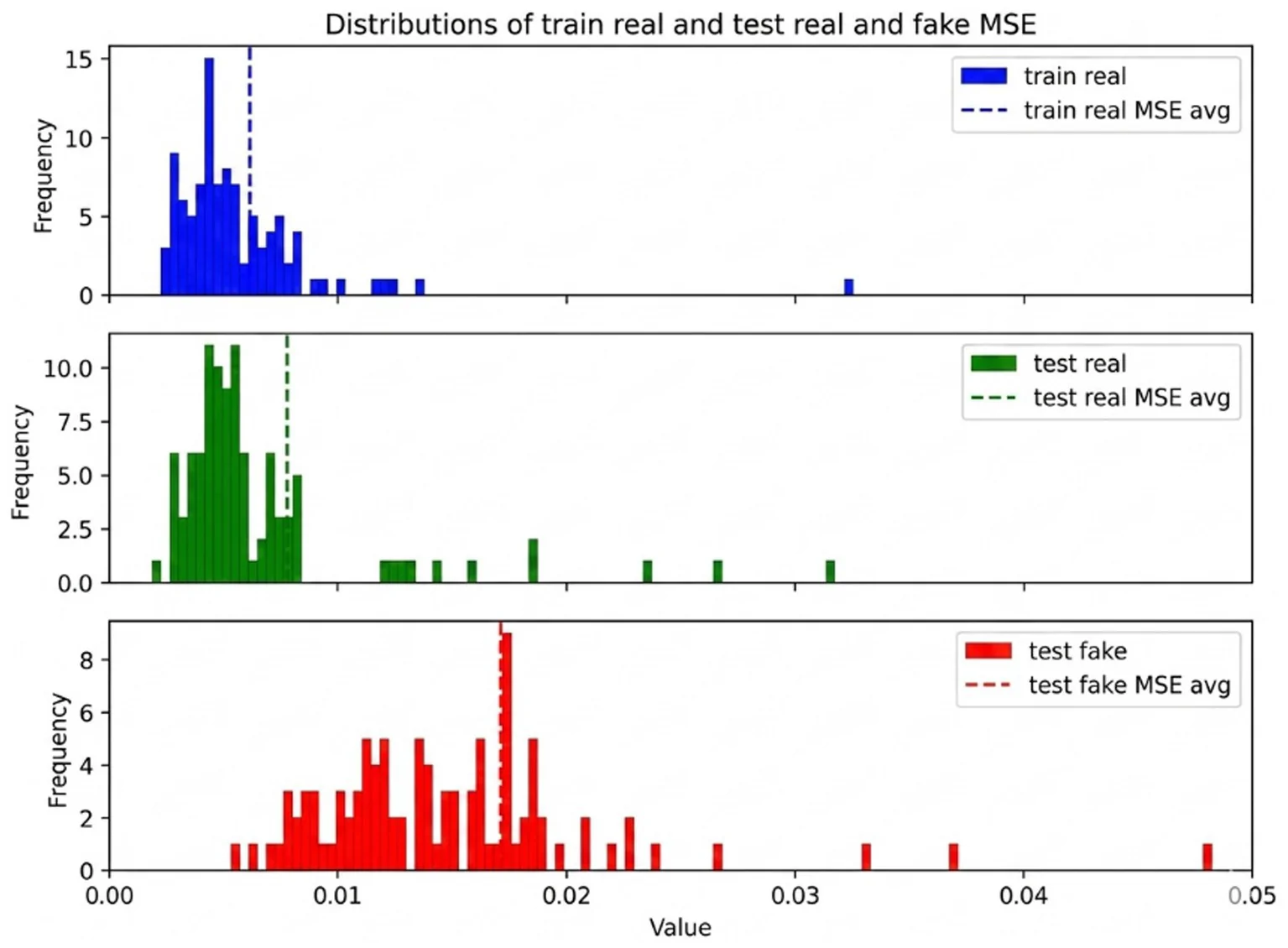
MSE distributions of 100 training real (blue), testing real (green) and testing fake (red) images.

Khalid and Woo (Khalid and Woo, 2020) also mention how only reconstruction loss may not be enough as an anomaly metric. Hence another metric is added to make the model more robust. KDE (Cao et al., 2016) is a non-parametric method to estimate the probability density function of a dataset. KDE can be used as a method to differentiate between normal and anomaly latent spaces. KDE is applied to estimate the probability density function (PDF) of the data. KDE smooths the density distribution by placing a kernel function at each data point and summing their contributions. Once the PDFs of the normal and anomaly latent spaces are estimated using KDE, the density values at specific points or regions can be compared. Areas of high density in the normal latent space are expected to represent the typical behavior of the data, while deviations from this behavior may indicate anomalies.

The equation for KDE is given by Equation 4. Here *n* is the number of data points, *x_i_* are the data points, *K* is the kernel function used and *h* is the bandwidth to which *K* is scaled. The Gaussian kernel function and a bandwidth of 1.0 is used in CAE-DAD. The equation for the Gaussian kernel function K, is given by Equation 5. Here x represents the distance from the center of the kernel. While the latent space spans 4,096 dimensions, its true intrinsic dimensionality is much lower because the autoencoder compresses real faces into a tight, dense cluster. In this compact space, a Gaussian kernel with a bandwidth of (*h* = 1.0) smoothly traces the boundaries of genuine data without hiding deepfake anomalies. This natural distribution directly justifies our log-density threshold of −7,500, which serves as a highly effective baseline filter to improve anomaly separation.

$$\hat{f}(x)=\frac{1}{n}\sum_{i=1}^{n}K_{h}(x-x_{i})$$ (4)

$$K(x)=\frac{1}{\sqrt{2\pi }}e^{-\frac{x^{2}}{2}}$$ (5)

### 3.6 Threshold selection

There can be multiple ways of choosing a threshold, including manually choosing it based on the distribution (Siddalingappa and Kanagaraj, 2021), choosing a particular interquartile range (Khalid and Woo, 2020). For the MSE threshold selection, a few methods were tested, the equations for which are given below. Table 1 shows the comparison between the accuracy achieved using these threshold selection methods. Only the MSE metric is used for classification here, without KDE, so the contribution of MSE can be observed. As evadible from the results, the method of choosing the *mean MSE + mean absolute deviation (MAD) MSE* gives the best standardizable results, without the need for manual selection by a human, and this is further proved in the K-fold cross-validation in 5.2. Equations 6–8 show the three methods of threshold selection.

$$\mathrm{MS}E_{\text{threshold}}=\mathrm{MS}E_{\text{mean}}$$ (6)

$$\mathrm{MS}E_{\text{threshold}}=\mathrm{MS}E_{\text{mean}}+\mathrm{MS}E_{\text{standard deviation}}$$ (7)

$$\mathrm{MS}E_{\text{threshold}}=\mathrm{MS}E_{\text{mean}}+\mathrm{MS}E_{\text{mean absolute deviation}}$$ (8)

**Table 1:** Accuracy achieved by various MSE threshold selection methods.

Method	Accuracy
Equation 6	0.83
Equation 7	0.56
Equation 8	0.89

To further confirm that this method produces the desired threshold, the MSE distributions of the testing real and testing fake images are taken, and the threshold which will give the best classification accuracy is found. To do this, Youden’s J statistic is used. We create a uniform list of samples separated by 0.0001 from the minimum value of the combined two distributions and the maximum value. Then for each sample the sensitivity and specificity (Vujovic, 2021) and the J value is calculated, the equation for which is given below. The sample with the best J value is the best threshold. Youden’s J statistic is a common metric used to find a threshold as it is immune to class imbalance and considers both sensitivity and specificity. The equation for finding J is given by Equation 9.

J=Sensitivity+Specificity−1 (9)

The best threshold found by this method is 0.0083, and the accuracy achieved by using this threshold is 0.90. By using the chosen method from Equation 8, the threshold value comes out be 0.0086, and the accuracy as 0.89. Hence it can be noted that the chosen method is close to perfect. Now, one might wonder why we cannot just use this J statistic method of choosing our threshold, that is because here we know which data is real and fake, and our just using it to confirm our hypothesis. In the real world, the data will not be labelled, hence we cannot use any information gathered from testing data to build our model.

For the KDE threshold, the threshold is simply set to −7,500, as from all the tests conducted, it was concluded that most of the real images sampled lie between 0 and −7,500. This works well enough and gives us an increase in accuracy over using just MSE.

### 3.7 Classification

For the final classification a combination of MSE and KDE are used so that there is redundancy and at least one of the metrics catches the deepfake. First the images are classified using just MSE, then using just KDE, and then a logical OR operation is performed to choose the final prediction. Logical OR is chosen as if either metric identifies the image as a deepfake, it should be classified as such. If logical AND is chosen, then both the models would have to identify the image as fake for it to be classified as such. Choosing logical OR prioritizes recall over precision, as a deepfake being classified as real is more damaging than real data being classified as fake.

## 4 Experimental set-up

### 4.1 Dataset

The Real and Fake Face Detection Dataset Real and Fake Face Detection (2019) by Yonsei University is used for this research. It is publicly available on Kaggle. It contains both, real and fake face images of size 600×600. The fake images are expert-generated high-quality photoshop images. The fake images are divided into three categories, easy, medium and hard. There are 1,081 real images and 960 fake images in the dataset. We ensure a proportionate amount of easy, medium and hard level of deepfakes are included in the testing set. The test-train split is kept at 80/20. This dataset is used as it is small and easy to train models with on limited hardware. The dataset also contains deepfakes that range from easy, medium and hard to detect, ensuring the model is tested thoroughly. The creators of the dataset believe the images are high quality photoshops which can be harder to detect than AI generated deepfakes which tend to have patterns that classifiers learn. The Human-Indistinguishable Deepfake Dataset (HiDF) (Kang et al., 2025) was used as an additional dataset for cross dataset validation. This dataset contains 62,000 images out of which we have used 200 real images and 200 fake images for the testing purpose. The model was trained using the Yonsei dataset and tested using the HiDF dataset as well.

### 4.2 Experimental test bed

All the work is done on a 2021 MacBook Pro with an M1 Pro chip with 16GB RAM. The OS used is MacOS Ventura 13.1. The Python version used is 3.9.13. The Keras and TensorFlow version used is 2.7.0. The NumPy version used is 1.22.4.

## 5 Results and discussions

### 5.1 Model performance

The training loss curve of the model is shown in Figure 6. As evident in the graph, the model achieves convergence after epoch 140. The final training loss of the model is 0.0047. This means it can reconstruct real images well. The model is benchmarked with standard performance metrics like accuracy, precision, recall, F1 Score, Cohen’s Kappa Score and Area Under the ROC Curve (AUC) (Vujovic, 2021). The equations for the same are given below, from Equations 14–19.

**Figure 6:**
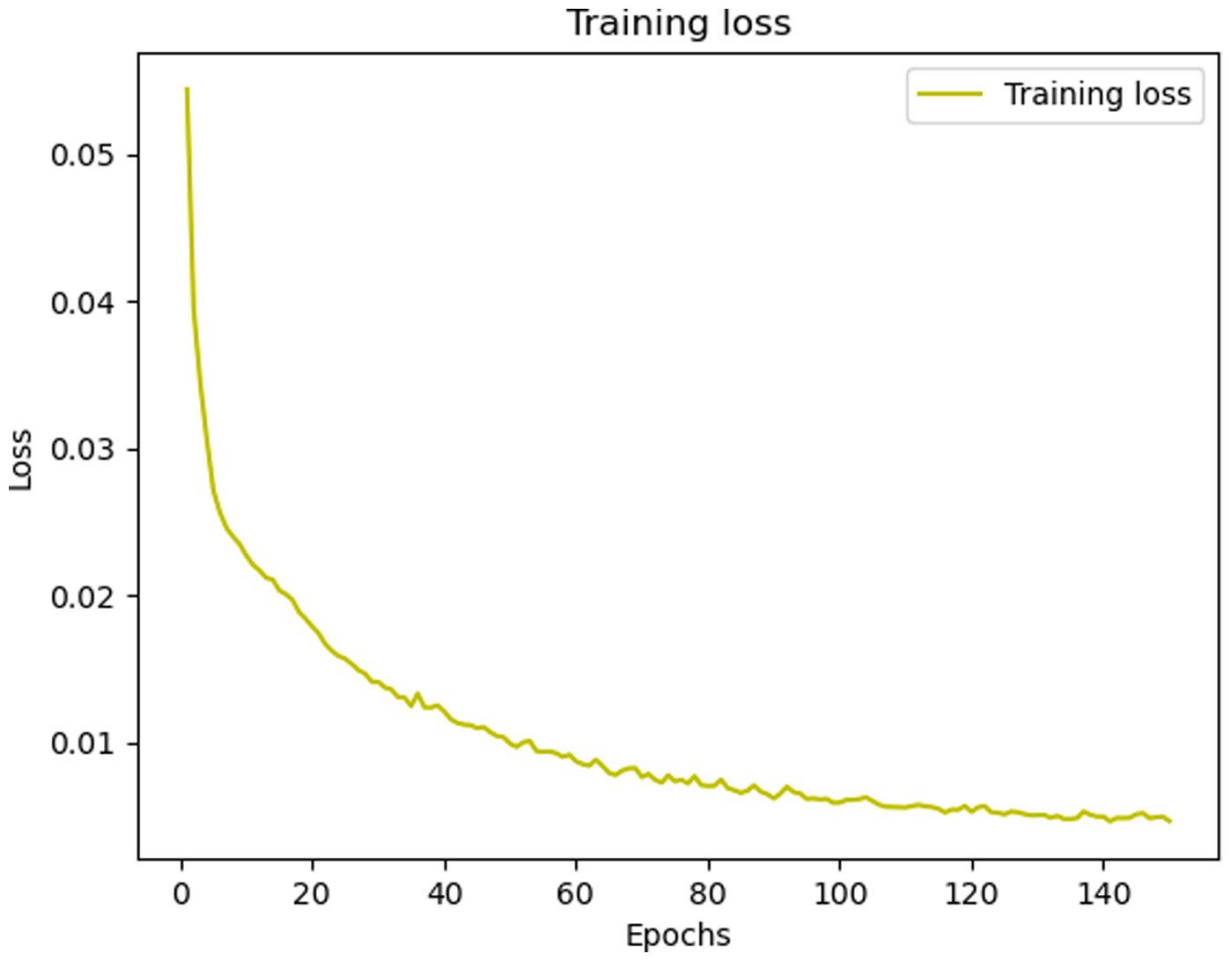
Training loss curve of the model.

Equations 10–14 are for the True Positive (TP), False Positive (FP), True Negative (TN) and False Negative rates (FN). These are required for the calculation of further metrics. TP (True Positives) is the number of correctly predicted positive samples. TN (True Negatives) is the number of correctly predicted negative samples. FP (False Positives) is the number of incorrectly predicted positive samples. FN (False Negatives) is the number of incorrectly predicted negative samples. In this case, the positive sample would be a fake image, while the negative sample would be a real image.

$$\mathrm{TP}=\sum_{i=1}^{n}1(\text{actua}l_{i}=1\;\text{and predicte}d_{i}=1)$$ (10)

$$\mathrm{TN}=\sum_{i=1}^{n}1(\text{actua}l_{i}=0\;\text{and predicte}d_{i}=0)$$ (11)

$$\mathrm{FP}=\sum_{i=1}^{n}1(\text{actua}l_{i}=0\;\text{and predicte}d_{i}=1)$$ (12)

$$\mathrm{FN}=\sum_{i=1}^{n}1(\text{actua}l_{i}=1\;\text{and predicte}d_{i}=0)$$ (13)

Accuracy is the ratio of correctly predicted samples to the total number of samples (Equation 14). Precision is the ratio of true positive samples to the total number of samples predicted as positive (Equation 15). Recall or specificity is the ratio of true positive samples to the total number of actual positive samples (Equation 16). F1 score is the harmonic mean of precision and recall, combining both measures into a single value (Equation 17). The Cohen’s Kappa score, often referred to as Kappa, is a statistic that measures the agreement between two annotators on a classification problem (Equation 18). It considers how much better the agreement is over what would be expected by chance alone. The Area under the Receiver Operating Characteristic (ROC) Curve (AUC-ROC) is a metric used to evaluate the performance of a binary classification model (Equation 19). It represents the area under the ROC curve, which plots the True Positive Rate (TPR) against the False Positive Rate (FPR) at various threshold settings. Figure 7 shows the Confusion Matrix and Figure 8 shows the ROC curve for the classification. The values for accuracy, precision, recall, F1 Score, Cohen’s Kappa Score and Area Under the ROC Curve (AUC) are given in Table 2.

$$\text{Accuracy}=\frac{\mathrm{TP}+\mathrm{TN}}{\mathrm{TP}+\mathrm{TN}+\mathrm{FP}+\mathrm{FN}}$$ (14)

$$\text{Precision}=\frac{\mathrm{TP}}{\mathrm{TP}+\mathrm{FP}}$$ (15)

$$\text{Recall}=\frac{\mathrm{TP}}{\mathrm{TP}+\mathrm{FN}}$$ (16)

$$F1=2\times \frac{\text{Precision}\times \text{Recall}}{\text{Precision}+\text{Recall}}$$ (17)

$$\kappa =\frac{2\times (\mathrm{TP}\times \mathrm{TN}-\mathrm{FP}\times \mathrm{FN})}{\left(\mathrm{TP}+\mathrm{FP})\times (\mathrm{FP}+\mathrm{TN})+(\mathrm{TP}+\mathrm{FN})\times (\mathrm{FN}+\mathrm{TN}\right)}$$ (18)

$$\mathrm{AUC}-\mathrm{ROC}=\int_{0}^{1}\mathrm{TPR}(\mathrm{FPR})\;d(\mathrm{FPR})$$ (19)

**Figure 7:**
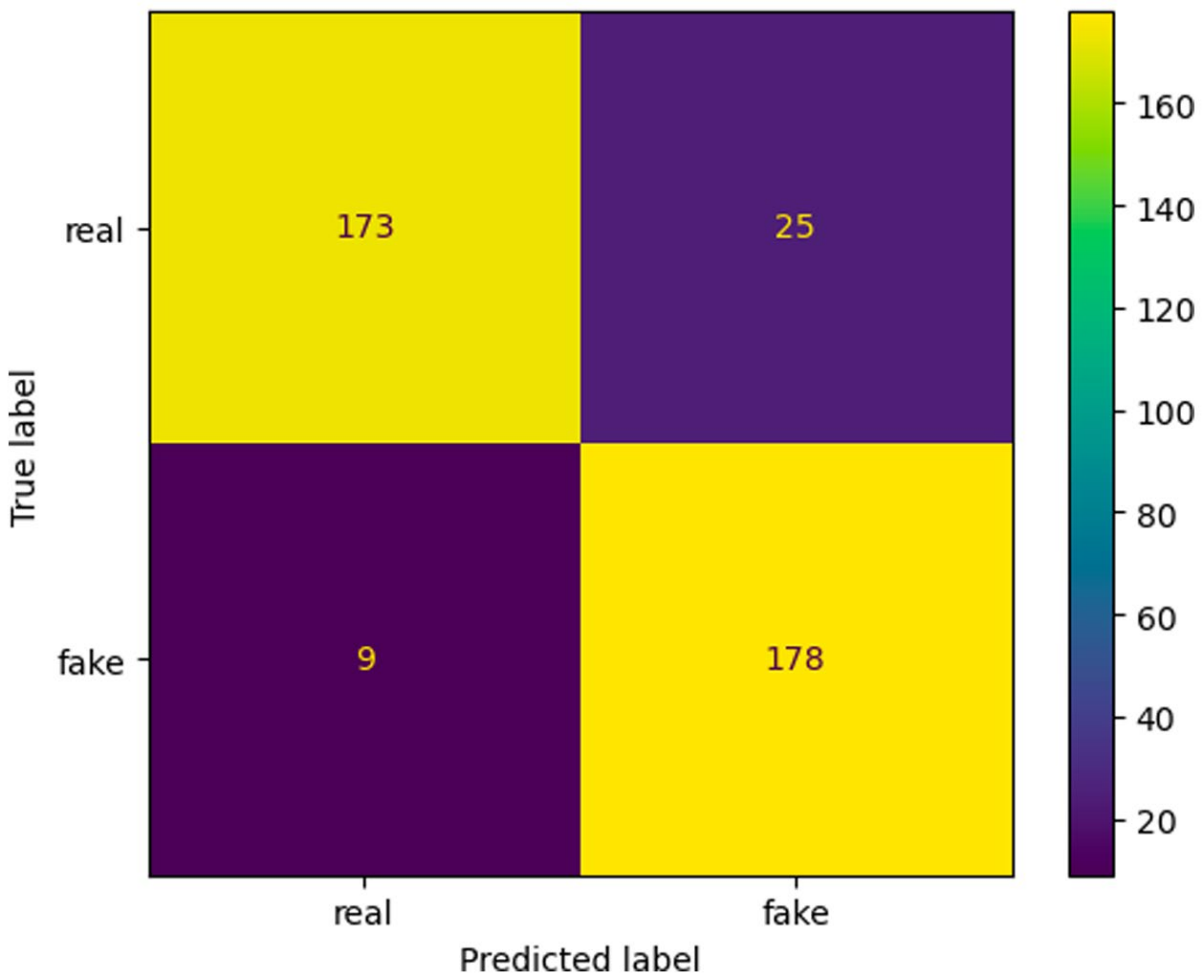
Confusion matrix for the classification model.

**Figure 8:**
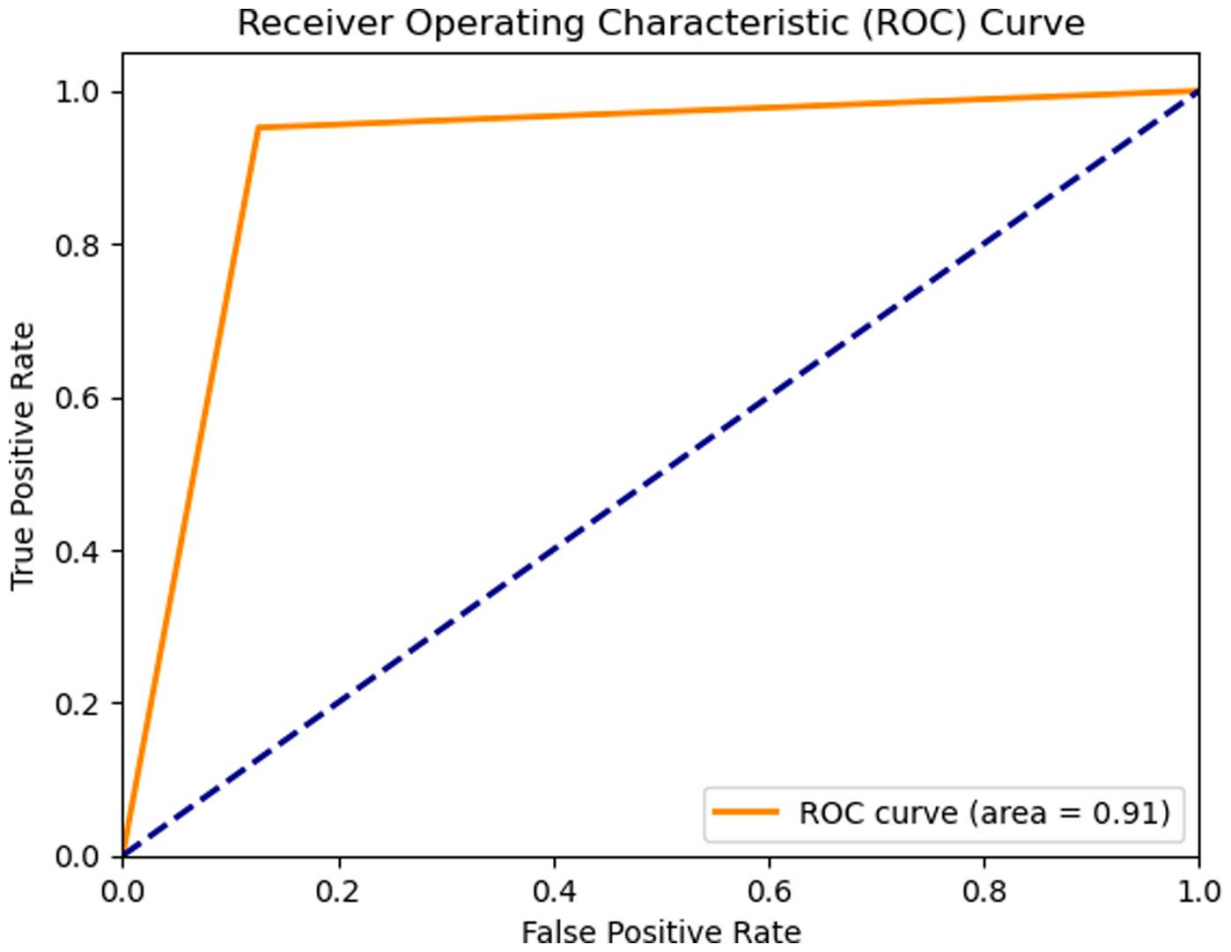
ROC curve for the classification.

**Table 2:** Performance metrics of the classification.

Datasets	Accuracy	Precision	Recall	F1 score	Cohen’s Kappa score	AUC
Yonsei dataset	0.91	0.87	0.95	0.91	0.82	0.91
HiDF dataset	0.85	0.8	0.82	0.79	0.71	0.8

### 5.2 K-fold cross-validation

K-fold cross-validation allows for robust model evaluation by partitioning the dataset into K subsets, training the model K times using K-1 subsets as training data and the remaining subset as validation data, and averaging the performance metrics across all K iterations, reducing the risk of overfitting, and providing more reliable estimates of model performance. 5-fold cross validation is performed on the model to validate it. The MSE threshold is chosen as the mean + MAD of the MSE distribution of the real training images. The KDE threshold is kept as −7,500. We ensure a proportionate amount of easy, medium, and hard fake images are present in each test set. The results are shown below, all values are rounded to 2 decimal places. Table 3 shows the performance metrics like accuracy, precision, recall and F1 score for each fold of the k-fold cross-validation test. Table 4 shows the mean and standard deviation for the metrics across the test. Based on the results and the size of our dataset, it can be said our model is generalized and the deviations across the test are within an acceptable range.

**Table 3:** Performance metrics of each fold in the 5-fold cross-validation test.

Fold	Training loss	Accuracy	Precision	Recall	F1 Score
Fold 1	0.0047	0.91	0.88	0.95	0.91
Fold 2	0.0048	0.88	0.86	0.89	0.88
Fold 3	0.0050	0.86	0.79	0.97	0.87
Fold 4	0.0048	0.89	0.82	0.98	0.90
Fold 5	0.0052	0.90	0.83	0.99	0.91

**Table 4:** Mean and standard deviation of performance metrics across the 5-fold cross-validation test.

Metric	Mean	Standard deviation
Training loss	0.0049	0.0002
Accuracy	0.89	0.017
Precision	0.84	0.031
Recall	0.96	0.036
F1 Score	0.89	0.016

Unlike the primary MSE threshold which utilizes an automated statistical formula, the secondary KDE threshold is structurally fixed at −7,500. Because high-dimensional log-density values introduce severe numerical instability into standard automated optimization metrics like Youden’s J-statistic, a data-driven baseline approach was required. This boundary was empirically established by mapping the stable lower-bound log-density distribution of authentic training samples. Functioning as a rigid secondary filter for structural anomalies, this fixed boundary catches extreme latent deviations that escape the MSE threshold, optimizing the overall framework accuracy from 89 to 91%.

### 5.3 Comparative analysis

It would be interesting to see what the results would be if we changed some parts of the model. It would also demonstrate the value of the modules present in the system. For all the comparisons, the models are trained with the same parameters as mentioned before, for 150 epochs on the dataset with an 80/20 train-test split.

VGG16 is swapped as the base encoder model with other standard CNN models like ResNet50 (He et al., 2016), Xception (Chollet, 2017) and MobileNet (Howard et al., 2017) to see the difference in performance. Figure 9 shows the training loss achieved with different base encoder models. Vgg16 based model has the lowest training loss, but the other three are also close. It must be noted the performance of MobileNet is impressive considering how light weight it is compared to the other models. The performance benefit of using RetinaFace to crop the face out of images is also tested. Figure 10 shows the training loss achieved with and without RetinaFace cropping. As evadible from the graph, adding the RetinaFace cropping has a significant positive impact on the ability of the model to reconstruct faces. The loss decreases by almost 33% after implementing the cropping method to add attention to the model.

**Figure 9:**
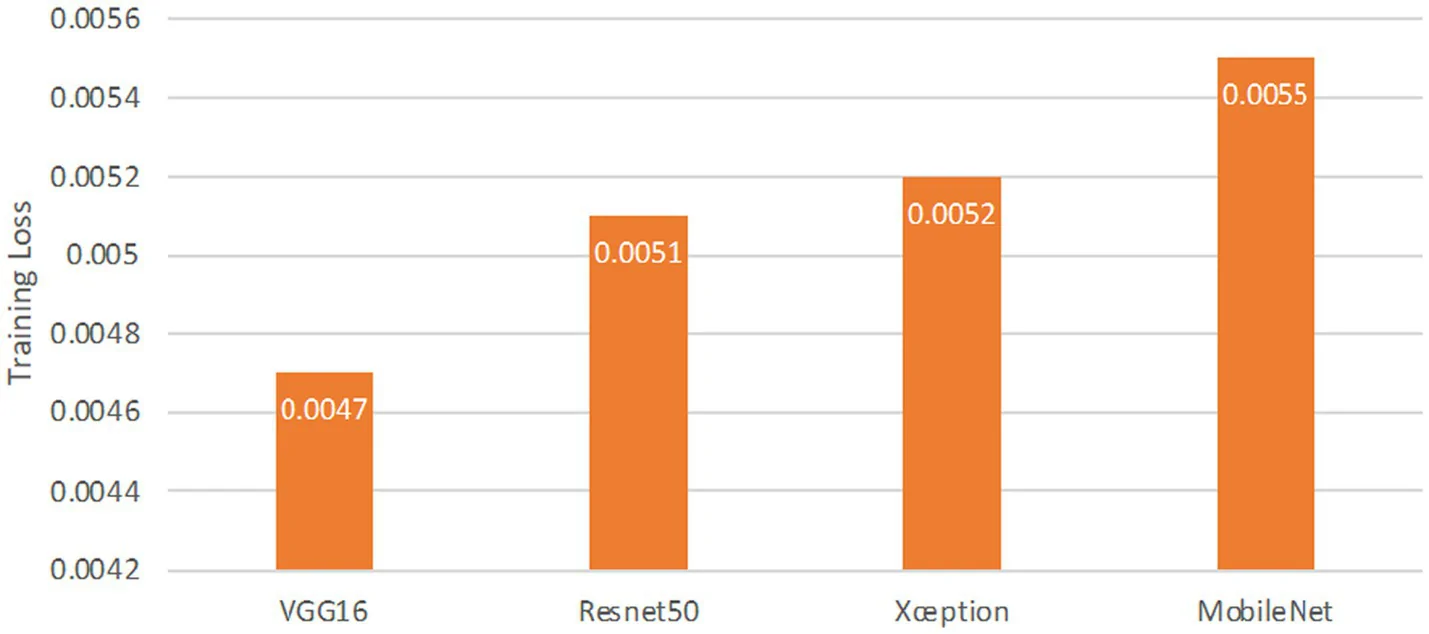
Training loss with various encoder base models.

**Figure 10:**
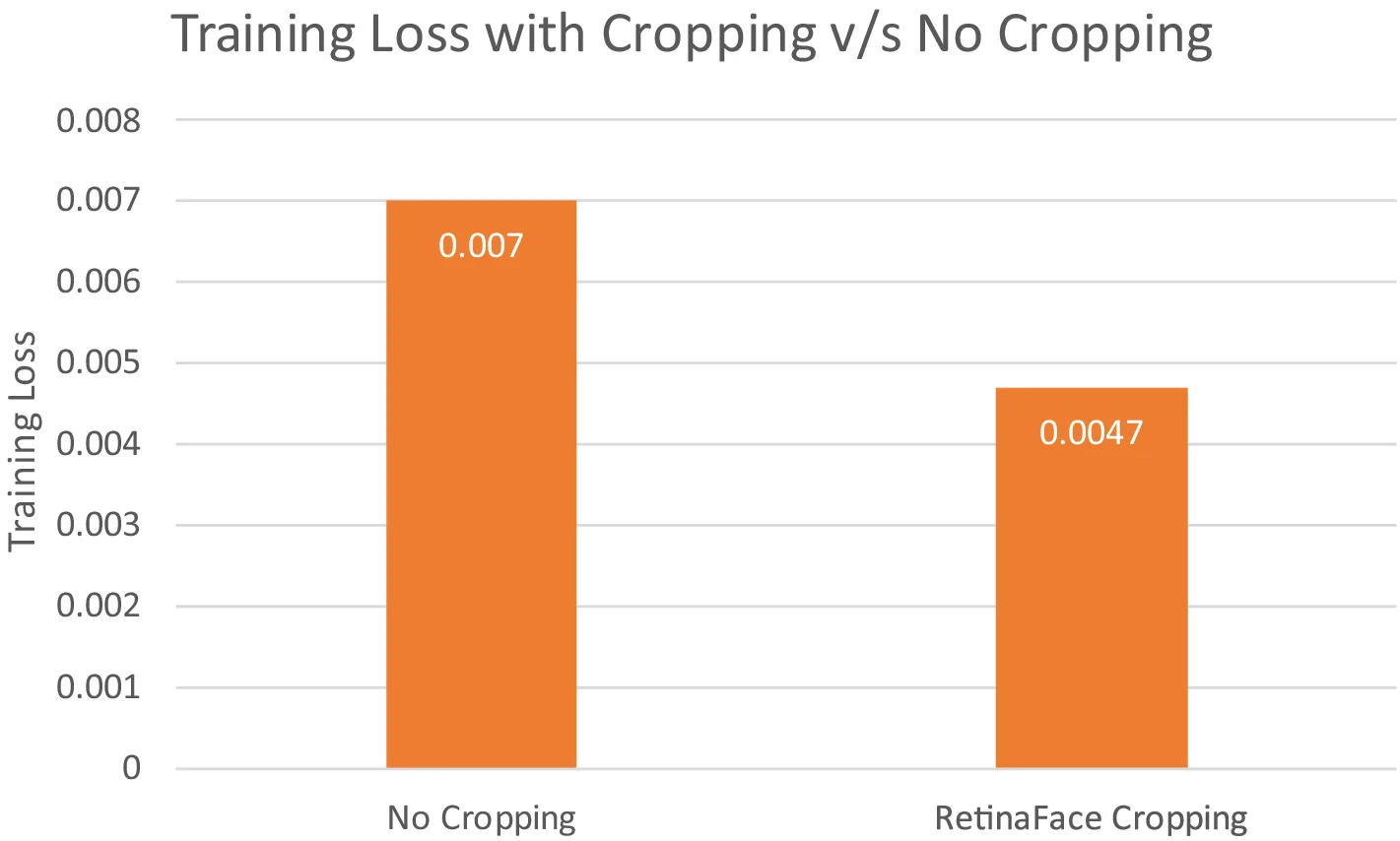
Training loss with and without RetinaFace cropping.

### 5.4 Comparison with existing models

CAE-DAD is compared with existing models on the same dataset. All the models have been tested on the same dataset to draw a more accurate comparison between them, and find out where CAE-DAD stands amongst them. Table 5 shows the accuracy of CAE-DAD compared with other state of the art models. The row highlighted in bold is the proposed model. As evident from the results, CAE-DAD is better than or on par with most of the existing models and only slightly worse than the DFP method from (Raza et al., 2022). These results can be considered a success as the existing models are all CNN architectures trained on both real and a type of fake images and may not be as generalizable. Apart from this, Rana et al. (2022) finds that on average deep learning models achieve an accuracy of 89.73%. The accuracy achieved by CAE-DAD is in line with this.

**Table 5:** Performance of CAE-DAD compared with state of the art models.

Model	Accuracy
NAS-Net (Raza et al., 2022)	0.83
Xception (Raza et al., 2022)	0.84
MobileNet (Raza et al., 2022)	0.88
VGG16 (Raza et al., 2022)	0.90
DFP (Raza et al., 2022)	0.94
ResNet18 + KNN (Rafique et al., 2023)	0.90
GoogLeNet + KNN (Rafique et al., 2023)	0.81
ELA + VGG16 (Qurat-ul-ain et al., 2021)	0.64
ELA + VGG19 (Qurat-ul-ain et al., 2021)	0.61
CAE-DAD (Proposed)	0.91

### 5.5 Discussion

There are a few things which reduce the quality of the model which should be addressed. The presence of young children’s or baby photos in the dataset, decreases the quality of it, as the slightly different features of young children’s faces dilute the adult features. The probability of a small child being deepfaked are low. Accessories like hats, glasses, jewelry can also lead to false classification, because as far as the model is concerned it is an anomaly. The same can be said to hand gestures covering the face. These are some of the things which can lead to a lower precision value, as real images are classified as anomalies, or fakes. Because CAE-DAD is a one-class anomaly detector optimized for standard adult facial geometry, contextual elements like children’s unique proportions, accessories, or hand gestures are mathematically flagged as structural deviations. Since the dataset lacks standardized metadata to partition samples into explicit difficulty tiers, these specific physical occlusions explain the slight inflation in our global false positive rate. Future work will incorporate robust feature regularization to better distinguish between benign physical occlusions and malicious digital synthesis. While evaluated on expert manual composites rather than AI-synthesized imagery, the proposed methodology success demonstrates a robust capability to detect structural and geometric deviations from genuine facial features. Because it operates as a one-class anomaly detector trained exclusively on authentic faces, it identifies the boundary of “realness” itself rather than overfitting to specific GAN or diffusion artifacts. These results validate the framework’s core utility against high-quality digital forgeries, establishing a strong foundation for future testing on generative AI datasets.

## 6 Conclusion and future work

The convolutional autoencoder based deepfake detection model has been successfully built and is on par or better than existing state of the art models. The results demonstrate the capability of CAE-DAD to effectively reconstruct facial images, enabling the detection of anomalous patterns inherent in deepfake content. By leveraging latent representations learned during training and the reconstruction MSE loss, our model effectively distinguishes between genuine and manipulated images, achieving promising results in terms of accuracy and robustness. CAE-DAD achieves respectable performance and lays the ground work for more deepfake detection models to use this autoencoder anomaly detection approach rather than training the CNN models on both real and fake data. CAE-DAD achieves an accuracy of 91% of the real and fake faces dataset. It has also been compared with other state of the art methods and performs better than most of them. By leveraging the power of deep learning and innovative architectural designs, we take a step forward in enhancing the trustworthiness and integrity of digital media in an era of increasing misinformation and manipulation.

In the future, there are a few things which can be done to take CAE-DAD even further. First would be training the model on a bigger dataset, with more diversity in the facial attributes like skin colour, gender, age, eye colour etc. This will ensure a more generalized model that is not biased towards any particular attribute. One could also try converting the architecture into a variational autoencoder to see if the latent space distribution helps with classification.

## Data Availability

Publicly available datasets were analyzed in this study. This data can be found at: the Real and Fake Face Detection Dataset by Yonsei University is used for this research. It is publicly available on Kaggle - https://www.kaggle.com/datasets/ciplab/real-and-fake-face-detection.
